# Increased Bone Marrow Adiposity in a Context of Energy Deficit: The Tip of the Iceberg?

**DOI:** 10.3389/fendo.2016.00125

**Published:** 2016-09-16

**Authors:** Olfa Ghali, Nathalie Al Rassy, Pierre Hardouin, Christophe Chauveau

**Affiliations:** ^1^Laboratoire de Physiopathologie des Maladies Osseuses Inflammatoires, Université de Lille, Boulogne-sur-Mer, France; ^2^Laboratoire de Physiopathologie des Maladies Osseuses Inflammatoires, Université du Littoral Côte d’Opale, Boulogne-sur-Mer, France

**Keywords:** bone marrow adiposity regulation, osteoporosis, gelatinous bone marrow transformation, mesenchymal stem cell differentiation, anorexia nervosa

## Abstract

Elevated bone marrow adiposity (BMA) is defined as an increase in the proportion of the bone marrow (BM) cavity volume occupied by adipocytes. This can be caused by an increase in the size and/or number of adipocytes. BMA increases with age in a bone-site-specific manner. This increase may be linked to certain pathophysiological situations. Osteoporosis or compromised bone quality is frequently associated with high BMA. The involvement of BM adipocytes in bone loss may be due to commitment of mesenchymal stem cells to the adipogenic pathway rather than the osteogenic pathway. However, adipocytes may also act on their microenvironment by secreting factors with harmful effects for the bone health. Here, we review evidence that in a context of energy deficit (such as anorexia nervosa (AN) and restriction rodent models) bone alterations can occur in the absence of an increase in BMA. In severe cases, bone alterations are even associated with gelatinous BM transformation. The relationship between BMA and energy deficit and the potential regulators of this adiposity in this context are also discussed. On the basis of clinical studies and preliminary results on animal model, we propose that competition between differentiation into osteoblasts and differentiation into adipocytes might trigger bone loss at least in moderate-to-severe AN and in some calorie restriction models. Finally, some of the main questions resulting from this hypothesis are discussed.

## Introduction

The bone marrow (BM) is predominantly composed of two fractions, namely the hematopoietic and stromal fractions. Mesenchymal progenitor cells reside in the stromal fraction of the BM and can differentiate primarily into osteoblasts and adipocytes (1).

Bone marrow adiposity (BMA) is defined as the proportion of the BM cavity volume occupied by adipocytes. A rise in the BMA can be caused by an increase in the size and/or number of adipocytes (2). BMA levels differ for males vs. females; they are usually higher in women than in men, and higher in female animal models than in males (3). It is also well known that BMA increases with age in a bone-site-specific manner. At birth, the BM is largely hematopoietic (red marrow) but is gradually converted to fatty (yellow) marrow over the lifespan (4–7). The age-related progression of BMA in the axial skeleton differs from that observed in the appendicular skeleton. The fat fraction in iliac crest biopsies is relatively low during childhood but increases gradually with age (8). In contrast, BMA in long bones of the limbs starts to rise during the first few years of life, with progressive colonization of the medullar cavity from the diaphysis. This process is complete at the age of 25 years or so (9).

These observations suggest that (i) the presence of adipocytes in the BM is a physiological, age-related phenomenon, and (ii) these adipocytes may have positive effects. In fact, adipocytes can supply energy for the high level of bone remodeling required during puberty (10, 11). Furthermore, BM adipocytes may be involved in thermogenesis and heat dissipation because they display some of the characteristic features of brown adipocytes (12). However, an increase in BMA may be linked to certain pathophysiological situations. In fact, osteoporosis or compromised bone quality related to mechanical unloading (13), aging (14), menopause (15), diabetes (16, 17), obesity (18, 19), or anorexia nervosa (AN) (20) is frequently associated with high BMA [for a review, see the article by Hardouin et al. (21) in this special issue].

Two hypotheses have been proposed to explain the bone alterations induced by BMA increase: (i) the elevated BMA observed in osteoporotic patients is caused by a shift in mesenchymal stem cell commitment from the osteogenic pathway to the adipogenic pathway at the expense of bone formation (22–24) and (ii) BM adipocytes can regulate the BM microenvironment and act negatively on the balance between bone formation and bone resorption (11, 25–27) and hematopoiesis (28–31).

A better understanding of the quantitative and/or qualitative changes in BM adipocytes during osteoporosis might enable the development of novel therapeutic strategies. Of the various pathologic contexts associated with osteoporosis, AN is of particular interest. Although osteoporosis in AN has been extensively described, the present article discusses changes in BMA and the regulation of this process in a context of energy deficit and, then, proposes some perspectives for research in this specific field.

## BMA and Energy Deficit

Anorexia nervosa is a major public health concern; it is the psychiatric disease with the highest mortality rate. Given that AN often coincides with puberty, this condition markedly interferes with bone mass gain and has a long-term impact on bone quality (32). In particular, studying AN may reveal novel, hitherto unsuspected links between BMA and bone mass. First, secondary osteoporosis in AN is often associated with an increase in BMA that contrasts with a decrease in the adiposity of other fatty tissues (Table 1). Second, some studies (but not others) have found that levels of inflammation markers are not elevated in patients with AN (despite amenorrhea) (33, 34). Given that most patients with AN have a low bone mass, inflammation-independent mechanisms may be involved in this bone loss.

**Table 1:** **Variations in BMA and the presence of gelatinous BM transformation in patients with AN, as reported in case–control studies and case reports**.

	*N*	Age	BMI	BMA measurement site	Method for measuring BMA	Change in BMA	Presence of GBMT

	Anorexic/normal	Anorexic/normal	Anorexic/normal				
Bredella et al. (35)	14/12	29.5 ± 7.1/30.8 ± 6.6	17.7 ± 1/22.1 ± 1.7	Femoral diaphysis	(1)H-MRS	↑	ND
Fazeli et al. (36)	7/15	33.1 ± 2.8	18.2 ± 0.6/21.9 ± 0.4	Lumbar vertebra	(1)H-MRS	↑	ND
				Proximal femoral epiphysis		→	
				Proximal femoral metaphysis		→	
				Proximal femoral diaphysis		→	
Ecklund et al. (37)	30/–	16.1 ± 1.6/16.3 ± 1.6	16.9 ± 1.5/22.3 ± 2.0	Distal femoral metaphysic	MRI and relaxometry	↑	ND
				Proximal tibia metaphysis		↑	
Bredella et al. (20)	10/10	29.8 ± 7.6/30.8 ± 6.6	17.6 ± 1/21.9 ± 1.7	Lumbar vertebra	(1)H-MRS	↑	ND
				Proximal femoral epiphysis		→	
				Proximal femoral metaphysis		↑	
				Proximal femoral diaphysis		↑	
Mayo-Smith et al. (38)	15/58	15–33/18–44	ND/ND	Lumbar spine (L1-L4)	Dual energy CT scanning	↑	ND
Geiser et al. (39)	20/19	15–56/21–56	14.5/22	Femoral epiphysis	(1)H-MRS and relaxometry	↓	ND
				Femoral diaphysis		→	
				Lumbar spine		→	
Abella et al. (40)	44/–	22.5 ± 5.3/–	ND/–	Iliac crest	Histology	↑ in 35% of cases	In 50% of cases
Boutin et al. (41)	10/–	17–57/–	ND/–	Many different bone sites	MRI	ND	In all cases
Lambert et al. (42)	10/19	17.2 ± 0.7/18.7 ± 0.5	14 ± 0.5/22.3 ± 0.4	Lumbar spine, pelvis, proximal femur	MRI	ND	40% of cases
Vande Berg et al. (43)	19/–	15–35/–	–/–	Proximal to distal lower limb	MRI	ND	79% of cases
Vande Berg et al. (44)	14/–	27 ± 10/–	13.9 ± 2.6/–	Lumbar spine, pelvis, proximal femur	MRI	ND	43% of cases
Mant and Faragher (45)	6/–	16–44	ND	Iliac crest	Histology	ND	83% of cases
Case reports of AN with GBMT^a^	18		12.1 ± 1.5/9.3–16	Iliac crest for half of the cases. Proximal femur, pelvis, foot or not specified for the other cases	Histology, cytology^b^	↓	All cases

*BMI, body mass index; BMA, bone marrow adiposity; GBMT, gelatinous bone marrow transformation; H-MRS, proton magnetic resonance spectroscopy; ND, not determined; MRI, magnetic resonance imaging; CT, computed tomography; ND, not determined; AN, anorexia nervosa*.

*Arrows: the variations in patients with AN differed significantly from those observed in control subjects*.

*^a^Data from 15 case reports on GBMT in patients with AN (46–59)*.

*^b^Except for two cases where MRI was applied*.

However, the fact that a link between energy deficit and increased BMA is not always observed raises questions as to the relevance of BMA in AN. Indeed, several studies of osteoporotic patients with AN did not observe elevated BMA (Table 1). In fact, the studies having found an increase in BMA tended to feature patients with a higher body mass index (BMI) than those in studies that did not observe this increase. Moreover, a number of studies that categorized patients into BMI classes highlighted a relationship between the severity of body weight loss and changes in the BMA (40, 43, 44). Interestingly, Abella et al.’s histological study of patient biopsies described a hypoplastic BM in which an increase in both the fat fraction and adipocyte diameter was associated with moderate body weight loss (40). The researchers also observed areas of unaffected or hypoplastic BM and focal gelatinous degeneration in patients with intermediate levels of body weight losses (40). Last, the most severe cases featured a high microadipocyte count in a hyaluronic acid matrix. It is noteworthy that, in one of the patients in Abella et al.’s study, the BM’s appearance normalized after the resumption of food intake and the return to an appropriate bodyweight (40). Vande Berg et al. used magnetic resonance imaging to highlight the presence of serous-like BM at different skeletal sites in anorectic patients (44). They distinguished between two groups as a function of the presence (group A) or absence (group B) of a water-like pattern in the marrow spaces. The individual BMI values in the group A were all under 13.5 kg/m^2^, whereas six of the eight patients in group B had a BMI above 15 kg/m^2^. Abella et al. did not find an intergroup difference in disease duration, which suggests that body weight loss is a key factor in BM alterations (39–41, 46).

This short overview of the relationship between BMA alterations and the severity of the body weight loss highlights the complexity of the processes leading to these alterations and probably, thereafter, to bone loss. Studies of animal models should provide some clues to these processes. In rodent models of food restriction or calorie restriction, only four published studies have focused on BMA (Table 2). First, Hamrick et al. studied the effects of a 40% food restriction in 14-week-old male mice (60). The 10-week protocol led to a body weight loss of 30% (relative to control mice) and the total disappearance of BM adipocytes in the distal femur and lumbar vertebra (60) (two adipocyte-poor medullar sites). Despite this lack of adipocytes, the cortical thickness fell in the femur and lumbar vertebra, and the trabecular thickness fell in the femur (60). However, Devlin et al.’s 2010 study of the effects of a 9-week, 30% food restriction in 3-week-old male mice highlighted decreases of 11 and 27% in the bone volume/total volume (BV/TV) ratio and in cortical thickness, respectively (61). Although this protocol induced a relative body weight loss of 40% (compared with control mice), the food-restricted animals were nevertheless 80% heavier at the end of the protocol than at the start. The main impact on bone microarchitecture was observed in the femur, where the BM adipocyte density was 700% higher than in control animals. Third, Baek and Bloomfield fed 6-month-old Sprague-Dawley female rats a calorie-restricted diet for 12 weeks (62). At the end of this period, the calorie-restricted rats displayed body weight losses of 20% (relative to day 0) and 25% (relative to control rats). The volumetric bone mineral density of the proximal tibia was 14% below that of control mice, while the BMA in the proximal femur had doubled. More recently, Cawthorn et al. showed that 6 weeks of a 30% calorie-restricted diet in 9-week-old female mice induced a final increase in tibia BMA of 700% vs. mice able to feed *ad libitum* (27). In our separation-based model of AN (SBA), 8-week-old female mice were housed singly and submitted to time-restricted feeding (in order to avoid a compensatory increase in food intake and, thus, attain a daily food intake close to that of *ad libitum* mice) (63). The calorie-restricted animals rapidly lost around 25% of their initial body weight, which corresponded to a body weight loss of 40% (relative to control mice) after 10 weeks of the protocol. The bone mass gain observed in control mice was curtailed after 2 weeks of the protocol (63). After 10 weeks, the tibia trabecular BV/TV ratio, trabecular thickness, and cortical thickness were, respectively, 32, 31, and 15% lower in SBA mice than in control mice. However, the BMA level in the proximal tibia was similar to that observed in control mice (unpublished data). These results showed (as did Hamrick et al.’s study) that energy deficit can induce bone alterations in the absence of an obvious increase in BMA (60). Interestingly, Cawthorn et al. showed very recently that, in rabbit, CR leads to bone loss, even without increases in BMA (64).

**Table 2:** **Variations in BM adipose tissue (BMAT) content in calorie-restricted rodent models**.

Characteristics of the model	Period of protocol	BW	Bone	BMAT content	Reference
Male mice 40%, food restriction	From 14 to 24 weeks of age	−30% vs. *ad libitum*	Low cortical and low trabecular thickness (femur)	−100% (distal femur)	Hamrick et al. (60)
Male mice 30%, calorie restriction	From 3 to 12 weeks of age	−40% vs. *ad libitum* +80% vs. day 0	Low cortical thickness and low trabecular BV/TV (femur)	+700% (distal femur)	Devlin et al. (61)
Female rats	From 6 to 9 months of age	−25% vs. *ad libitum* −20% vs. day 0	Low trabecular volumetric bone mineral density but non-significant changes in cortical bone (tibia)	+100% (proximal femur)	Baek and Bloomfield (62)
Female mice, 30% calorie restriction	From 9 to 15 weeks of age	−23% vs. *ad libitum* −3% vs. day 0	Low trabecular thickness and low cortical volume (tibia)	+700% (tibia, above fibula junction)	Cawthorn et al. (27)
Female mice, time-restricted feeding	From 8 to 18 weeks of age	−40% vs. *ad libitum* −25% vs. day 0	Low trabecular BV/TV and thickness, low cortical thickness (tibia)	Non-significant (proximal tibia)	Zgheib et al. (63) and unpublished data

To learn more from these animal datasets about BMA regulation, one can attempt to compare them. The five above-mentioned studies assessed rodents of different genders and at different ages. The well-known differences between male and female physiology and the differences in BMA variation with age make it difficult to compare males with females (65). For the studies of male animals, the difference in observed BMA changes between Hamrick et al.’s study and Devlin et al.’s study might be linked to the age difference. One might conclude that calorie restriction induces a large increase in BMA in young male rodents only. However, it is also noteworthy that there is a late-onset BMA increase in male control mice; this might reduce the possible BMA increase in older animals (as observed at the end of Hamrick et al.’s study). When considering the three studies of female rodents, Baek’s work was performed on rats aged up to 9 months. It is difficult to compare old female rats with the 8- and 9-week-old C57Bl/6 female mice used by Cawthorn et al. and Zgheib et al., respectively. The main differences consisted in the body weight changes (relative to the start of the study) and the BMA changes in the tibia. Cawthorn et al. observed a stable body weight and a 700% increase in BMA, whereas Zgheib et al. observed a 25% decrease in body weight but no significant change in BMA. Given that these two models were similar enough to enable a valid comparison, one can hypothesize that the magnitude of body weight loss influences the change in BMA most strongly in young adult female mice.

## Potential Regulators of BMA in a Context of Energy Deficit

As described above, it appears that BMA may be modulated by age, gender, and the severity of the energy deficit. However, it is important to bear in mind that BMA can also be affected by many different biological factors, including hormones, growth factors, and pharmacologic agents. Although the molecular mechanisms involved in BMA regulation have not been fully defined, they appear to converge on the modulation of peroxisome proliferator activated receptor gamma 2 (PPARγ2) expression and/or activity.

When considering potential regulators of BMA in subjects with a severely reduced calorie intake, it has been shown that dysregulation of the growth hormone insulin-like growth factor 1 axis and low leptin levels can stimulate BM adipogenesis (19, 61, 66). Moreover, starvation prompts the mobilization of fat stores, which might activate PPARγ2 and stimulate adipocyte differentiation in the BM (27). Furthermore, calorie restriction in a mouse model resulted in low expression and activity of runt-related transcription factor 2 (Runx2) and tafazzin (TAZ), both of which are pro-osteogenic transcriptional factors in BM stromal cells (67, 68).

Several other investigators have established links between BMA and pre-adipocyte factor 1 (Pref1). Indeed, circulating Pref1 concentrations are higher in women with AN than in healthy controls (66). In women who have recovered from AN, Pref1 levels are significantly lower than in normal-weight controls (66). Hence, it is possible that elevated Pref1 levels in AN may be involved in the increase in BMA.

It is also known that the production of reactive oxygen species (ROS) is greater in AN patients than in control subjects (69). ROS greatly influence the generation and survival of bone cells (70). *In vitro* studies of bone metabolism have shown that oxidative stress inhibits osteoblastic differentiation and induces apoptosis (71, 72).

Interestingly, women with AN have higher cortisol levels than healthy controls (73, 74). Cortisol levels are also predictive of a low bone mineral density in AN (73). In animal models, Cawthorn et al. showed that both BMA and glucocorticoids increase during CR in mice, but neither of these increases during CR in rabbits, suggesting a narrow link between these two phenomena (64). Experiments *in vitro* suggest that corticosteroids lead to adipogenesis by activating PPARγ2 and blocking the Wnt signaling pathway (75, 76). These findings also suggest that cortisol, Pref1, and leptin are all potential BMA regulators in a context of energy deficit.

Furthermore, women with AN have low estrogen levels (77, 78). Postmenopausal estrogen deficit has been linked to high BMA (79). *In vitro* studies have demonstrated that estradiol induces a pro-osteogenic lineage shift in BM stromal cells, whereas estrogen deficit in aging mice was found to induce a decrease in the expression and activity of the pro-osteogenic, anti-adipogenic factor sirtuin type 1 (80, 81). One can, thus, conclude that estrogen is likely to be involved in the regulation of BMA.

Moreover, it is important to note that BM adipose tissue (BMAT) expansion contributes significantly to the elevated serum adiponectin levels and skeletal muscle adaptation observed during CR (27). Indeed, the study by Cawthorn et al. suggested that BMAT is a major source of circulating adiponectin in states of leanness, and that (through endocrine functions) BMAT can have extraskeletal, systemic effects (27). Thus, adiponectin may also regulate BMA in this context.

Last, it is probable that ongoing and/or future research will prompt us to take account of new factors that regulate adiposity in a context of energy deficit.

## Perspectives

This short overview of the relationship between BMA alterations and the severity of body weight loss highlights the complexity of the underlying processes and the mechanism of subsequent bone loss.

With a view to designing BMA-focused therapeutic strategies against osteoporosis in a context of energy deficit, one major question is “*do BM adipocytes trigger bone loss in a context of energy deficit?*” Several studies of patients with AN have shown that average-to-severe body weight loss tends to lead to normal or decreased BMA. This suggests that (i) the BMA increase observed in patients with mild body weight loss may be a late marker of changes that occur in BM and that result in low bone mass, and (ii) the absence of an increase in BMA does not necessarily mean that the skeleton is healthy. Some studies of CR animal models have shown that bone quality can decrease when the BMA is stable or even when it falls. This finding suggests that (at least in these models) hypotheses based on the effect of factors released by mature adipocytes on the BM microenvironment are not the most realistic. If the latter phenomenon is nevertheless involved, it might have a secondary role in the downregulation of bone physiology. Thus, BM adipocytes might not trigger bone loss in moderate-to-severe AN and in some CR models. Next, hypotheses involving competition between differentiation into osteoblasts and differentiation into adipocytes should be considered. Interestingly, our analysis of the differentiation capabilities of BM stromal cells from SBA and control mice found that the SBA cells displayed a huge increase in adipogenesis at the expense of osteoblastogenesis (unpublished data). This was observed in SBA mice that displayed normal or low BMA, which led us to suppose that the BM of these mice contains many pre-adipocytes that are not able to synthesize and store lipids *in vivo*. These preliminary results strengthen the second hypothesis, although further experiments will be required to confirm these observations and to determine which factors in the culture medium trigger this pre-adipocyte maturation and adipocyte activity. The glucose concentration appears to be a relevant candidate factor. However, favoring hypotheses based on the importance of pre-adipocytes raises another question: “*Do pre-adipocytes influence their microenvironment?*” The characterization of stromal cells at the earliest stage in culture might help to answer this question.

If one acknowledges that a shift in the differentiation of BM stromal cells toward the adipocyte lineage is the main BM event during bone mass loss in a context of energy deficit, the next question would be “*how is this commitment regulated?*” One way of addressing this question would be to develop CR models with different levels of body weight loss. One could then compare BMA, bone quality/turnover, concentrations of circulating hormones known to have effects on adipogenesis and osteoblastogenesis, and the BM stromal cells’ differentiation capabilities.

Tools are already available for studying how the differentiation of BM stromal cells is reprogrammed in animal models of CR and how differentiation is linked to the associated low bone mass. However, the development of novel tools for the *in vivo* assessment of specific cell types in the BM (such as pre-adipocytes) remains a true challenge.

## Author Contributions

OG, NAR, PH, and CC discussed the concept, compiled the literature, and wrote the paper.

## Conflict of Interest Statement

The authors declare that the research was conducted in the absence of any commercial or financial relationships that could be construed as a potential conflict of interest.
